# Evaluating the Educational Video Materials for Radiation Education on Nursing Students and Nurses: A Quasi-Experimental Research

**DOI:** 10.3390/nursrep15050159

**Published:** 2025-05-02

**Authors:** Minoru Osanai, Yoshiko Nishizawa, Yuka Noto, Ryoko Tsuchiya

**Affiliations:** 1 Department of Radiation Science, Hirosaki University Graduate School of Health Sciences, Hirosaki 036-8564, Japan; ominoru@hirosaki-u.ac.jp; 2 Graduate School of Community Health Studies, Hirosaki University of Health and Welfare Graduate School, Hirosaki 036-8102, Japan; 3 Department of Nursing Science, Hirosaki University Graduate School of Health Sciences, Hirosaki 036-8564, Japan; noto@hirosaki-u.ac.jp (Y.N.); tsuchiya@hirosaki-u.ac.jp (R.T.)

**Keywords:** radiological nursing, nursing education, radiation education, radiation protection, educational material

## Abstract

**Background/Objectives**: Although medical radiation practice is essential for current medical care, many nursing students and nurses lack sufficient basic knowledge about radiation, and they are unfamiliar with learning about it. This study aimed to evaluate the usefulness of self-made video teaching materials for radiation education for nursing students and nurses after clarifying their basic knowledge of radiation. **Methods**: Educational video materials were developed to provide basic radiation knowledge. The video materials included scenes of radiation measurement, such as the attenuation of scattered X-rays with distance, and illustrations drawn by nursing students to make them familiar to nursing staff. This study included 29 nursing students and 16 nurses. The participants were instructed to answer 20 questions regarding the characteristics of radiation and its influence and protection measures. The same questions were asked again after watching the video materials. **Results:** Nursing students and nurses correctly recognized the classification of medical or occupational exposure and the three principles for reducing external exposure; however, it became clear that dose limits do not apply to medical exposure and that radiation units and their effects on the human body were not correctly recognized. Furthermore, the educational materials were effective because the scores and the percentage of correct answers increased after viewing the video materials. Furthermore, positive comments were expressed regarding the scenes of the experiments and the illustrations drawn by the students about the teaching materials. **Conclusions**: The contents that should be addressed more intensively were clarified, and the effectiveness of using video teaching materials in radiation nursing education was demonstrated.

## 1. Introduction

Diagnostic radiation procedures, such as computed tomography (CT) and interventional radiology, have advanced remarkably and become essential tools in modern medicine [1,2]. Nurses who assist with radiological examinations play an important role in providing appropriate medical care. Because medical staff, including nurses, are exposed to a non-negligible amount of scattered X-ray in radiation practices [3,4,5,6,7], they should have appropriate radiation knowledge and be properly protected from scattered X-ray while performing their procedures. The Fukushima Daiichi Nuclear Power Plant accident in Japan in 2011 triggered calls for increased emphasis on radiation education for nursing students and nurses [8,9]. Furthermore, it is widely known that radiation exposure increases health risks, such as cancer and cataracts [10]. Because the dose limits for the eye lens in occupational exposure have been significantly lowered based on recommendations from the International Commission on Radiological Protection (ICRP) [11,12,13,14], radiation education is becoming increasingly important to ensure appropriate protection for medical staff [15,16,17,18,19,20,21]. In this context, the Radiological Nursing Society of Japan released a model syllabus for radiation nursing in 2019 to promote radiation nursing education at educational institutions [22]. The model syllabus is available in two versions: a 15-class version and a 2-class shortened version. The syllabus covers the content required for the nursing profession (e.g., basic radiation knowledge, an overview of examinations and treatments using radiation, the basics of nursing care, and radiation protection measures). In contrast, the model syllabus may not be widely adopted because nursing schools are rarely equipped with radiation scientists or nursing faculty who have expertise in radiation. Furthermore, a survey of nursing faculty members revealed a lack of educational materials for radiation education [23]. Therefore, there is a need to develop video teaching materials about radiation that can be widely used in class. Furthermore, nursing students and nurses are often uncomfortable in the field of radiology science, which is closely related to scientific subjects such as physics and mathematics [24]. In addition, while radiation nursing education should be provided to both nursing students and nurses, the differences in the degree of their knowledge about radiation are not clear. Thus, issues remain regarding the promotion of radiation education.

In this study, trial video material designed by a radiation expert who is also involved in radiation nursing education, including the basics of radiation, radiation medicine, and radiation protection, was developed. This video material was developed with the aim of making it widely available. As one of its unique features, the teaching material also includes experimental scenes of radiation measurement to make them more approachable. A questionnaire survey on the characteristics of radiation, the effects of radiation on the human body, and radiation protection, among other topics, was conducted for nursing students and nurses before and after viewing the prototype video material. To contribute to the promotion of radiation nursing education, this study aimed to clarify the degree of knowledge that nursing students and nurses have regarding radiation and to evaluate the usefulness of the developed video materials. A questionnaire survey was conducted before viewing the video materials to evaluate the degree of basic knowledge regarding radiation among nursing students and nurses. The same questionnaire survey was administered after viewing the video materials to compare the results before and after viewing the video materials and assess their usefulness.

## 2. Materials and Methods

### 2.1. Development of Video Materials for Radiation Education

The concepts for developing the teaching materials were (1) to keep the content to the minimum necessary for easier understanding, (2) to include experimental scenes of radiation measurement that would be difficult for nursing faculty to prepare, and (3) to use illustrations created by nursing students to make the materials more approachable.

The video materials comprise (A.) Radiation Basics and Terminology, (B.) Three Principles for Reducing External Exposure, and (C.) Radiation Protection in Medicine. In particular, (A.) Radiation Basics and Terminology included radiation around us, the use of radiation, terms and units, half-life, and the types and characteristics of radiation. (B.) Three Principles for Reducing External Exposure included distance, time, and shielding, and (C.) Radiation Protection in Medicine included measures to reduce external exposure in medical radiological practice.

The text and illustrations for the educational materials were placed in PowerPoint 2021 (Microsoft Japan Co., Ltd., Tokyo, Japan), and narration was added. The data on the slides were then converted to video.

### 2.2. Outcome Measurements

#### 2.2.1. Research Design

A quasi-experimental study was conducted on nursing students and nurses, which included a fact-finding survey, a pre–post comparison test, and a comparison between groups.

#### 2.2.2. Participants

Subjects were selected through non-probabilistic sampling. Specifically, the participants were nursing students at two universities and nurses at hospital B in prefecture A in Japan. Leaflets were used to request cooperation for the survey, and registration for participation was conducted via Google Forms (Google Japan G.K., Tokyo, Japan). All nursing students and nurses who agreed to participate in the survey were included, regardless of the grade levels of the nursing students or the years of clinical experience of the nurses. Since this survey was conducted on the basis of the voluntary efforts of nursing students and nurses, it was expected that the sample size would be small. Therefore, the sample size was not defined in this study. Forty-five participants (29 nursing students and 16 nurses) participated in the survey. The numbers of nursing students were as follows: 10 first-year students, 7 second-year students, 6 third-year students, and 6 fourth-year students. The median years of nursing experience among the nurses was 9.5 years (range: 2–31 years).

#### 2.2.3. Survey Period

The survey period was from February 2024 to March 2024. The survey was conducted using the questionnaire survey method outlined in the research paper.

#### 2.2.4. Knowledge of Nursing Students and Nurses Regarding Radiation

The self-reported questionnaire comprised 20 questions related to radiation basics. Table 1 presents the specific questions. Since no questionnaire for assessing radiation knowledge has been established, the content of the questions was determined through consideration and review by the authors. For each question, participants were instructed to select one correct or incorrect answer from three or four options.

#### 2.2.5. Evaluation of the Usefulness of the Developed Video Materials

After the questionnaire survey indicated in Section 2.2.4, “Knowledge of nursing students and nurses regarding radiation”, was conducted, the participants were instructed to watch the prototype educational video materials. After viewing the video materials, the same questionnaire survey was administered to evaluate the educational effects of the videos.

The ratio of the total number of correct answers after the study to the total number of correct answers before the study (effective ratio for each subject [ERES]) was calculated for each participant. If the value of ERES is greater than 1.0, it indicates that the subject’s score increased after viewing the video materials. Furthermore, for each question, the percentage of individuals who changed to the correct answer after studying (effective percentage for each question [EPEQ]) was calculated. For instance, an EPEQ value of 100% means that all the subjects changed from incorrect to correct answers after viewing the video materials.

Furthermore, the participants were instructed to comment on the video materials in the form of free descriptions. Specifically, the subjects were asked to comment on (1) good points, (2) difficult points to understand, (3) points for improvement, and (4) others throughout the video materials.

#### 2.2.6. Statistical Analysis

The chi-squared test was used to assess differences of knowledge in subjects between nursing students and nurses. The differences in the number of correct answers before and after viewing the video materials were analyzed using a *t*-test. The differences in ERES between nursing students and nurses were also analyzed using a *t*-test. The software SPSS-J ver. 28.0 (IBM Japan Ltd., Tokyo, Japan) and Statcel ver.3 (OMS Publishing Co., Ltd., Tokyo, Japan) were used for statistical analysis. A difference with a *p*-value of <0.05 was considered significant.

**Table 1:** Questions asked in the questionnaire survey.

No.	Question	Option	Coverage in the Teaching Materials
1	<Natural radiation dose> How much is the annual exposure dose from natural radiation in Japan?	a. 1.1 mSv/y b. 2.1 mSv/y * c. 3.1 mSv/y	Yes
2	<Classification of medical exposure> Which exposure categories apply to the patient?	a. Medical exposure * b. Occupational exposure c. Public exposure	Yes
3	<Classification of occupational exposure> Which exposure categories apply to medical staff?	a. Medical exposure b. Occupational exposure * c. Public exposure	Yes
4	<Attenuation with distance> If the distance from the radiation source is doubled, how small is the exposure dose?	a. One-half b. One-third c. One-fourth * d. One-fifth	Yes
5	<Scattered X-ray dose during portable X-ray imaging> In portable X-ray examination, what is the minimum distance from the patient at which scattered X-rays pose no problem?	a. 0.5 m b. 1 m c. 2 m * d. 5 m	Yes
6	<Penetration of X-rays> Which materials are most penetrable by X-rays?	a. Metal b. Fat * c. Water d. Bone	No
7	<Types of ionizing radiation> Which option is not included in ionizing radiation?	a. X-ray b. Alpha ray c. Ultraviolet * d. Neutron	No
8	<Half-life of radionuclide> What is the half-life of ^131^I?	a. 6 h b. 13 h c. 8 days * d. 30 years	Yes
9	<Unit of radioactivity> What is the unit of radioactivity?	a. Sv b. Gy c. Bq *	Yes
10	<Magnitude relationship of tissue weighting factor> What is the highest tissue weighting factor in the 2007 recommendations of the ICRP?	a. Lung * b. Gonads c. Esophagus d. Brain	Yes
11	<Three principles of reducing external exposure> Which is an inappropriate principle among the three for reducing external exposure?	a. Keep distance from the radiation source b. Take time to care for the patient * c. Use shielding	Yes
12	<Characteristics of stochastic effects> Which answer is false regarding stochastic effects?	a. Threshold exists * b. The frequency of effects increases with increasing dose c. Dose does not affect severity d. Cancer and leukemia exist	No
13	<Characteristics of deterministic effects> Which answer is false regarding deterministic effects (tissue reaction)?	a. Threshold exists b. Frequency of effects increases with increasing dose c. Cataract appears as a late effect d. Genetic effects exist *	No
14	<Characteristic of radiation> Which answer is a false characteristic of radiation?	a. Directness b. Penetrating effect c. Not felt by the 5 senses d. Cannot be measured *	Yes
15	<Principle of radiation protection> Which option is not applicable to the principle of radiation protection?	a. Justification b. Optimization of protection c. Strictness * d. Application of dose limits	No
16	<Application of dose limits> Which category is not applicable to the concept of dose limits?	a. Medical exposure * b. Occupational exposure c. Public exposure	Yes
17	<Type of radiation source> Which option is not a source of radiation?	a. natural b. radioisotope c. X-ray tube d. MRI *	No
18	<How to wear a personal dosimeter> When you perform a radiation procedure while wearing an X-ray protector (i.e., lead apron), where will you wear your personal dosimeter?	a. Inside the protector b. Outside the protector c. Outside and inside the protector *	No
19	<Protective effect of X-ray protector> By wearing an X-ray protector, how much can you reduce exposure due to scattered X-rays?	a. 30% b. 50% c. 70% d. 90% or more *	Yes
20	<Patient dose in chest X-ray examination> What is the effective dose to the patient during a chest X-ray examination?	a. 0.06 mSv * b. 0.5 mSv c. 1.0 mSv d. 5.0 mSv	No

The “*” in the options indicates the correct answer. Because the actual survey was administered in Japanese, this table is presented in a provisional translation into English. Titles inside “< >” are summaries of the questions, which were not listed in the actual questionnaire.

## 3. Results

### 3.1. Development of Video Materials for Radiation Education

**Figure 1:**
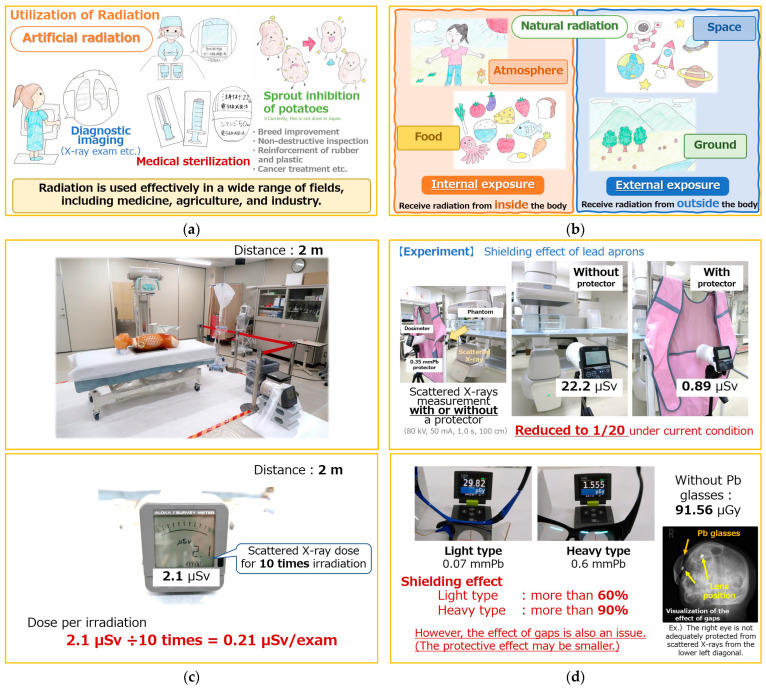
Examples of scenes in the video materials. (**a**) Examples of useful applications of radiation (e.g., diagnostic imaging, sterilization, and food irradiation). (**b**) Pathways of exposure from natural radiation, such as the atmosphere, food, space, and ground. (**c**) Scattered X-ray dose in portable radiography. (**d**) Shielding effect of protectors (“lead aprons”) and radiation protection glasses. Illustrations in the educational materials were drawn by nursing students. The actual teaching materials were produced in Japanese. These figures are presented in a provisional translation into English.

The length of the video materials was approximately 30 min. Figure 1 presents still images from the video materials. As scenes of measurement experiments, the educational materials included scattered X-ray doses during portable X-ray photography and the protective effects of personal protective equipment, such as protectors (“lead apron”).

### 3.2. Knowledge of Nursing Students and Nurses Regarding Radiation

Table 2 presents the percentage of correct answers to the 20 questions in the questionnaire survey. In addition to the percentage of correct answers, the table also shows the breakdown of the number of responses for each option, the chi-squared value, *p*-value, and phi coefficient (Cramer’s coefficient of association). The highest percentage of correct answers among all participants (both nursing students and nurses) was 97.8% for “Q2. Which exposure categories apply to the patient?” The next highest percentages of correct answers were 88.9% for both “Q3. Which exposure categories apply to medical staff?” and “Q11. Which is an inappropriate principle among the three for reducing external exposure?” In contrast, the question with the lowest percentage of correct answers was “Q16. Which category is not applicable to the concept of dose limits?” with 11.1% choosing the correct answer of “medical exposure”. The next question with the lowest percentage of correct answers was “Q9. What is the unit of radioactivity?” The correct answer of “Bq” was selected by 13.3% of all participants. Another question that also demonstrated a low percentage of correct answers was “Q12. Which answer is false regarding stochastic effects?”, with a correct response rate of 17.8%.

Three questions exhibited significant differences (*p* < 0.05) between nursing students and nurses. For “Q1. How much is the annual exposure dose from natural radiation in Japan?”, 69.0% of the nursing students and 25.0% of the nurses chose the correct answer, “2.1 mSv/y”. Moreover, in response to “Q8. What is the half-life of ^131^I?”, 62.1% of the nursing students and 18.8% of the nurses selected the correct answer, “8 days”. Furthermore, 3.4% of the nursing students and 31.3% of the nurses answered correctly—“Bq (becquerel)”—to “Q9. What is the unit of radioactivity?”

### 3.3. Usefulness of the Developed Video Materials

Scores before and after viewing the video material for each subject, where one correct answer to a question was counted as one point, are shown in Table 3. The average scores of the nursing students before and after watching the video were 10.2 and 14.0, respectively. Similarly, the mean scores of nurses before and after viewing the video were 10.1 and 14.1, respectively. Both nursing students’ and nurses’ scores after viewing the video material were significantly higher than those before viewing the video (*p* < 0.001). The maximum increase in score after viewing the video materials was 10 points, the mean increase was 3.9 points, and the minimum increase was 0 points.

**Figure 2:**
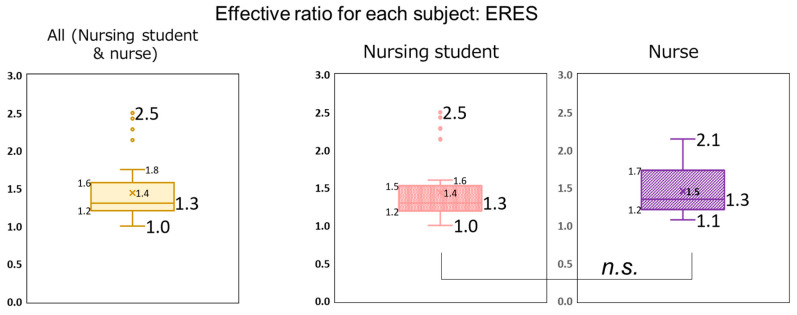
Distribution of the effective ratio for each subject (ERES) for all participants, nursing students, and nurses.

In this study, 95.6% of the participants exhibited an ERES > 1.0. This result indicates that the number of correct answers among almost all participants increased after viewing the educational video materials. Figure 2 presents the distributions of ERES among all participants, nursing students, and nurses. For all participants, the ERES ranged from 1.0 to 2.5 (median: 1.3). For nursing students, the ERES ranged from 1.0 to 2.5 (median: 1.3), and for nurses, the ERES ranged from 1.1 to 2.1 (median: 1.3). No statistically significant differences in ERES were observed between nursing students and nurses.

**Figure 3:**
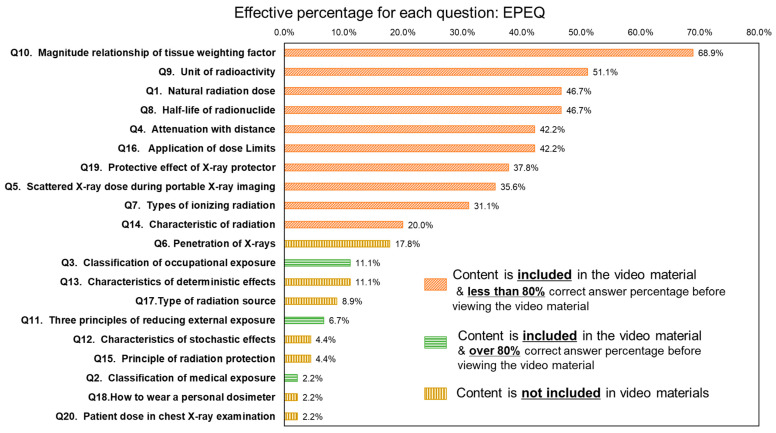
Effective percentage for each question (EPEQ). The bar graph indicates whether the content related to each question is covered in the teaching materials. Furthermore, it distinguishes questions that already had a high percentage of correct answers before the video was viewed.

Figure 3 presents the EPEQ values. The graphs also indicate whether the content related to each question is covered in the video materials. The questions with high EPEQ values among all participants were “Q10. What is the highest tissue weighting factor in the 2007 recommendations of the ICRP?” (68.9%) and “Q9. What is the unit of radioactivity?” (51.1%). The 10 items with the highest EPEQ values were questions regarding the content covered in the video materials. The EPEQ values of these items ranged from 20.0% to 68.9%. In contrast, the EPEQ values for questions related to content not addressed in the video materials were low, ranging from 2.2% to 17.8%. Among the questions addressed in the educational materials, the EPEQ values for those that had >80% correct answers before viewing the video materials ranged from 2.2% to 11.1%.

In the free comment question, a total of 64 and 43 responses were provided by nursing students and nurses, respectively. Table 4 presents examples of the comments made by participants in the free description section of the questionnaire survey. There were positive comments about the measurement experiments and hand-drawn illustrations included in the teaching video materials. In contrast, there were requests for simplification of the slides and for more detailed explanations. Furthermore, some comments called for improvements to maintain the participants’ concentration.

## 4. Discussion

Because of a lack of appropriate knowledge regarding radiation, some medical staff, such as nurses, are unnecessarily afraid of it [25,26,27]. To ensure that patients receive better medical care, nurses should first obtain adequate knowledge regarding radiation. Previous studies regarding awareness or knowledge of radiation among medical staff and students have highlighted the importance of radiation education [28,29,30,31,32]. In this study, to promote radiation nursing education, we developed video materials that could be easily understood by nursing students and nurses. Furthermore, after assessing the basic radiation knowledge of nursing students and nurses, the educational effectiveness of the self-made video materials was evaluated. The findings suggest that nursing students and nurses do not have sufficient knowledge of complex content related to radiation and that the video materials developed in this study are effective tools for learning.

In the knowledge survey of the nursing students and nurses regarding radiation, they demonstrated a clear understanding of the classification of radiation exposure (i.e., Q2: medical exposure and Q3: occupational exposure). Furthermore, they had a good understanding of the three principles for reducing external exposure (Q11). The percentage of correct answers to these questions among all participants was approximately >90%. Thus, it is assumed that current undergraduate and postgraduate education programs provide education on the classification of exposures and the principles of reducing exposure.

In contrast, as shown in the results of Q16 (correct answer percentage: 11.1%), because it is not accurately recognized that dose limits do not apply to medical exposure (i.e., patient exposure), the concept of “dose limits” in the basic principles of radiation protection should be adequately addressed in radiation education. Furthermore, the percentages of correct answers for Q9 (unit of radioactivity) and Q12 (characteristics of stochastic effects) were low. Because these contents are complex, continuous learning is essential. A previous study [33] has also reported that nurses do not have a high level of knowledge about radiation physics, the basic principles of radiation protection, and the stochastic effects of radiation. Furthermore, another previous study [28] also reported a lack of awareness of radiation effects among nurses. These points are consistent with our findings. It has been reported that nurses working in radiation practice are anxious about the uncertain effects of radiation exposure and that this may lead to a decrease in the quality of nursing care [27]. Thus, radiation education covering a wide range of fields must be provided on an ongoing basis. On the other hand, it has been reported that some nurses understand the principles of “justification”, such as “comparing risks and benefits”, which are included in the principles of radiation protection [34]. However, our survey results show that nurses do not have a high percentage of correct answers to the question about the principles of radiation protection (Q15). It is natural for the level of knowledge to vary among individual nurses. Therefore, it may be necessary to provide radiation education based on the knowledge level of the subjects.

Comparing the basic knowledge of nursing students and nurses regarding radiation, the knowledge level of nursing students was significantly higher than that of nurses in Q1 (natural radiation dose) and Q8 (half-life of radionuclide). In contrast, in Q9 (unit of radioactivity), the knowledge level of nurses was significantly higher than that of nursing students. This difference may be attributed to the relevance of the content of these questions to the nurses’ work. For instance, the natural radiation dose is not directly used in the nurses’ work; therefore, the percentage of correct answers was lower among nurses than among nursing students. Similarly, it is assumed that nurses had a higher percentage of correct answers for the question on units of radioactivity because the dosage of radioactive agents in nuclear medicine is expressed in Bq.

In the evaluation of the usefulness of the video materials, the majority of ERES scores were >1.0 (median 1.3) for nursing students and nurses, and the video materials were effective in radiation education. The 10 items with the highest EPEQ values were included in the educational video materials, suggesting that these materials are useful for radiation nursing education. In some cases, even items included in the educational video materials exhibited low EPEQ values. This occurred because the percentage of correct answers was high even before the video materials were viewed, which does not negate the usefulness of the video materials. The usefulness of digital education using video materials and other resources for radiation oncology has been reported [35]. Our study shows that video materials are also effective in basic radiation education for nursing students and nurses.

Free comments on the video materials showed that the participants positively evaluated the experimental scenes included in the teaching materials. Generally, teaching materials for radiation nursing education are limited, including scenes of radiation measurement experiments. This is a unique feature of our teaching materials. Another feature is the use of illustrations drawn by nursing students. We believe that this will be familiar to nursing students and nurses and will provide better educational benefits. In contrast, because there were some requests for improvements to the video materials, we have plans to develop a final version of the materials that addresses these points. Furthermore, we will develop advanced teaching materials on radiation protection measures in clinical practice.

## 5. Limitations

This study has some limitations that should be considered. It was shown that nursing students and nurses had a good understanding of the categories of radiation exposure and the three principles of reducing external exposure. However, because the universities to which the survey participants belong provide a certain level of radiation education, the participants’ knowledge of radiation may be higher than that of nurses and nursing faculty at other institutions. Therefore, a more detailed survey with a larger target population is required. In addition, because this survey was conducted voluntarily outside of class time or work by nursing students and nurses, the sample size was small. Therefore, generalization of the results from this study may be difficult. Next, because no comparisons were made with other video materials regarding their usefulness, the degree of usefulness of the video materials developed in this study is unclear. Furthermore, the ERES defined in this study does not reflect question-by-question changes due to studying, so questions that changed to incorrect answers after studying cannot be discussed. However, because the knowledge level of the participants regarding radiation increased after viewing the video materials, we believe that the video materials are useful as educational tools.

## 6. Conclusions

Both nursing students and nurses correctly understood the basics of exposure categories and principles for reducing external exposure but did not have sufficient knowledge regarding complex details such as the application of dose limits, units, and effects on the human body. After viewing the video materials developed in this study, the scores obtained in the questionnaire survey and the percentage of correct answers to each question increased, suggesting that the learning effect of the prototype teaching materials was high. Thus, this study clarified topics that should be addressed more intensively and demonstrated the effectiveness of using video teaching materials in radiation nursing education. We believe that it is important to continuously learn about basic radiation knowledge using effective teaching materials.

## Figures and Tables

**Table 2:** Percentage of correct responses to the 20 questions and its breakdown in the questionnaire survey.

		Number of Answers				Percentage of Correct Answers		χ2	*p*	φ/ Cramer’s V
		a	b	c	d		Total			
Q1 Natural radiation dose	Nursing student	7	20	2	-	69.0%	53.3%	8.12	0.017 *	0.43
	Nurse	10	4	2	-	25.0%				
Q2 Classification of medical exposure	Nursing student	28	0	1	-	96.6%	97.8%	0.56	0.45	0.11
	Nurse	16	0	0	-	100.0%				
Q3 Classification of occupational exposure	Nursing student	3	26	0	-	89.7%	88.9%	0.048	0.83	0.033
	Nurse	2	14	0	-	87.5%				
Q4 Attenuation with distance	Nursing student	9	2	18	0	62.1%	57.8%	0.735	0.69	0.13
	Nurse	7	1	8	0	50.0%				
Q5 Scattered X-ray dose during portable X-ray imaging	Nursing student	0	5	20	4	69.0%	62.2%	1.67	0.43	0.19
	Nurse	0	5	8	3	50.0%				
Q6 Penetration of X-rays	Nursing student	5	7	11	6	24.1%	20.0%	1.29	0.73	0.17
	Nurse	2	2	8	4	12.5%				
Q7 Types of ionizing radiation	Nursing student	1	2	18	8	62.1%	66.7%	1.15	0.77	0.16
	Nurse	0	1	12	3	75.0%				
Q8 Half-life of radionuclide	Nursing student	1	5	18	5	62.1%	46.7%	9.59	0.022 *	0.46
	Nurse	3	7	3	3	18.8%				
Q9 Unit of radioactivity	Nursing student	21	7	1	-	3.4%	13.3%	8.27	0.016 *	0.43
	Nurse	6	5	5	-	31.3%				
Q10 Magnitude relationship of tissue weighting factor	Nursing student	5	15	3	6	17.2%	17.8%	0.267	0.97	0.077
	Nurse	3	9	1	3	18.8%				
Q11 Three principles of reducing external exposure	Nursing student	2	25	2	-	86.2%	88.9%	1.18	0.56	0.16
	Nurse	1	15	0	-	93.8%				
Q12 Characteristics of stochastic effects	Nursing student	6	8	14	1	20.7%	17.8%	5.16	0.16	0.34
	Nurse	2	1	13	0	12.5%				
Q13 Characteristics of deterministic effects	Nursing student	1	6	4	18	62.1%	62.2%	4.26	0.23	0.31
	Nurse	1	0	4	10	62.5%				
Q14 Characteristic of radiation	Nursing student	6	2	6	15	51.7%	53.3%	0.812	0.85	0.13
	Nurse	3	2	2	9	56.3%				
Q15 Principle of radiation protection	Nursing student	15	0	13	1	44.8%	35.6%	3.76	0.15	0.29
	Nurse	11	0	3	2	18.8%				
Q16 Application of dose Limits	Nursing student	2	4	23	-	6.9%	11.1%	3.52	0.17	0.28
	Nurse	3	0	13	-	18.8%				
Q17 Type of radiation source	Nursing student	5	8	4	12	41.4%	55.6%	6.76	0.080	0.39
	Nurse	1	1	1	13	81.3%				
Q18 How to wear a personal dosimeter	Nursing student	6	6	16	-	55.2%	57.8%	0.553	0.76	0.11
	Nurse	4	2	10	-	62.5%				
Q19 Protective effect of X-ray protector	Nursing student	0	1	11	17	58.6%	62.2%	3.38	0.34	0.27
	Nurse	1	1	3	11	68.8%				
Q20 Patient dose in chest X-ray examination	Nursing student	10	12	5	2	34.5%	44.4%	3.85	0.28	0.29
	Nurse	10	4	2	0	62.5%				

The correct answer option in the number of answers column is underlined. In addition to the percentage of correct answers among all participants (nursing students and nurses), the percentages of correct answers among nursing students and nurses are also shown separately. The “*” in the *p*-value column indicates that there was a significant difference.

**Table 3:** Scores before and after viewing the video materials for individual nursing students and nurses.

Nursing Student			Nurse		
	Score			Score	
Subject	Before	After	Subject	Before	After
1	12	15	1	8	14
2	10	13	2	8	14
3	10	15	3	14	15
4	12	17	4	10	12
5	9	11	5	12	13
6	6	15	6	12	16
7	13	13	7	10	13
8	9	13	8	13	15
9	7	15	9	11	15
10	13	17	10	8	14
11	14	16	11	12	15
12	10	16	12	9	14
13	7	17	13	7	15
14	10	11	14	9	12
15	10	13	15	9	14
16	14	17	16	9	15
17	10	13			
18	11	14			
19	6	7			
20	12	14			
21	7	16			
22	8	11			
23	9	13			
24	11	11			
25	9	14			
26	11	14			
27	13	14			
28	12	15			
29	10	16			
Mean	10.2	14.0	Mean	10.1	14.1
*p*-value	<0.001		*p*-value	<0.001	

**Table 4:** Example of participants’ free comments on the educational materials.

Comment (Partial Excerpt/Summary)
<Good point>
-The video and examples of experiments were very easy to understand. -The scenes of experiments were interesting. -The handwritten illustrations were warm and friendly. -The actual irradiation scene was easy to visualize without portable X-ray equipment. -There were many explanations with diagrams; therefore, concepts were easy to understand visually.
<Difficult point to understand>
-The amount of information contained in one slide was excessive, and understanding the important points was difficult. -The explanations were too simple, and I could not understand them.
<Points to be improved>
-I need more detailed explanations in some parts. -I want to see a separate video for each section. -I think that it would be better if there was a quiz in the middle section to prevent sleepiness.

## Data Availability

Data addressed in this study can be requested from the authors of the article.

## Public Involvement Statement

There was no public involvement in any aspect of this research.

## Guidelines and Standards Statement

This manuscript was drafted against the Strengthening the Reporting of Observational Studies in Epidemiology (STROBE) statement.

## Abbreviations

The following abbreviations are used in this manuscript:

CT	Computed tomography
EPEQ	Effective percentage for each question
ERES	Effective ratio for each subject
ICRP	International Commission on Radiological Protection
